# Commentary: Physical Exercise as Personalized Medicine for Dementia Prevention?

**DOI:** 10.3389/fphys.2019.01358

**Published:** 2019-10-31

**Authors:** Thomas Gronwald, Henning Budde

**Affiliations:** ^1^Department of Performance, Neuroscience, Therapy and Health, Faculty of Health Sciences, Medical School Hamburg, University of Applied Science and Medical University, Hamburg, Germany; ^2^Faculty of Human Sciences, Medical School Hamburg, University of Applied Science and Medical University, Hamburg, Germany

**Keywords:** disease prevention, physical exercise, physical training, individualization, dosage

In their recently published perspective paper entitled “Physical exercise as personalized medicine for dementia prevention?” Müller et al. (2019) addressed and discussed the challenging issue of individualization of physical exercise and/or physical training in order to maximize its effects for dementia prevention. For this purpose, the following questions were addressed: “*(1) Which factors cause the large interindividual heterogeneity in response to physical training? (2) Are all outcomes affected equally by the individual responsiveness? and (3) How can we overcome non-responsiveness so that (almost) all individuals experience benefits?”*

Since relatively little attention has been paid to exercise prescription as a modifiable factor to increase the effects of physical interventions for disease prevention, the authors of this comment support the main idea of Müller et al. (2019) to intensify the efforts that aim to individualize physical interventions. However, while we believe that this perspective was well-intentioned, we believe that the salient aspects require further clarification.

(i) Firstly, in Müller et al. (2019) the terms “physical activity,” “physical exercise,” and “physical training” are used without a clear differentiation between their meanings. This becomes particularly apparent and confusing in the recommendation section as the authors use three distinct terms in five sentences (“*personalized preventive exercise strategies,” “personalized exercise programs,” and “personalized exercise training program”*). An appropriate use of terminology is mandatory and helps to avoid ambiguity, especially as the terms that are used represent different constructs (Budde et al., 2016a). In general, “physical activity” is defined as muscle-induced bodily movement which increases energy expenditure from 1.0 to at least 1.5 MET (Metabolic Equivalent of Task; Budde et al., 2016a). “Physical exercises” is characterized as specific, planned, and structured forms of physical activity. Physical exercises should be distinguished into acute physical exercise (single bout) and chronic physical exercise (repeated bouts of acute physical exercise; Budde et al., 2016a). Furthermore, chronic physical exercises can be denoted as “physical training” when it is conducted regularly in a planned, structured, and purposive manner with the objective to increase (or maintain) individual capabilities in one or multiple fitness domains (Budde et al., 2016a). Based on the facts that prevention constitutes a long-term (lifelong) and regularly conducted intervention strategy, and that individualization should be a planned and structured approach, we suggest that “training” is, in most circumstances, the most appropriate term to describe individualized physical training programs to prevent dementia and other (neurological) diseases. Please note that in this commentary, we use “physical intervention” as an umbrella term encompassing both “physical exercise” and “physical training.”

(ii) Secondly, Müller et al. (2019) explained the large interindividual heterogeneity of adaptations in response to physical interventions mainly with the non-modifiable factor “genetics.” It is unquestioned that the analysis of the individual genetics (e.g., the genome) helps to determine the potential magnitude of responses (e.g., be a potential responder or non-responder; respectively an individual which “did not respond” to a specific intervention, according to Pickering and Kiely, 2019) which, in turn, contributes to the understanding of interindividual heterogeneity in distinct outcome variables (Hawley, 2008; Lightfoot, 2008; Pescatello, 2008). However, while physical interventions influence epigenetic factors (e.g., transcription), the genome, as a non-modifiable factor, is not changed by physical interventions (Kaliman et al., 2011). Hence, the individualization of physical interventions relies on the modification of exercise prescription (as a modifiable-exercise-related factor) in order to account for the influence of non-modifiable factors (e.g., the genome) and modifiable non-exercise-related factors, including sleep, nutrition, and social and cognitive activities or stress (Sparks, 2017; Herold et al., 2019a; Pickering and Kiely, 2019); these are also crucial factors in adaptive responses (Horwitz et al., 2013; Peck, 2018) and dementia prevention (Müller et al., 2017; Kivipelto et al., 2018). In addition, the different capacity level (including level of performance) in various age groups should be considered to compare the effects of exercise interventions from a neurobiological perspective (Budde et al., 2018). Indeed, the modification of exercise prescription lead to changes in the dose provided by the physical interventions. Müller et al. (2019) stated that “*…the non-responder status can be mitigated by increasing the exercise intensity and/or dose…*”. However, a clear definition of dose is missing, and a detailed examination of how physical interventions should be individualized remains elusive in the presented perspective. In our opinion and to more effectively prompt the direction of the individualization of physical interventions, characterizing and operationalizing the dosage is imperative. More precisely, physical exercise variables could be objectified by indicators of external load, as work completed by the individual (e.g., external resistance, power, speed as a function of distance and duration, movement frequency, and mode or type of exercise with different muscle involvement and biomechanical load), independent of internal characteristics. Furthermore, environmental factors, such as climatic conditions, equipment, and the ground condition, also influence the external load. Finally, indicators of internal load, as individual and acute physiological, psychological, and/or biomechanical responses to the external load during physical exercise, and the influencing factors could be also used to objectify physical exercise variables (Impellizzeri et al., 2019). However, given that physical exercise variables, such as exercise intensity, can be operationalized using either measures of external load (e.g., running with a speed of 10 km/h) or internal load (e.g., running with 70% of maximum heart rate), the current definitions of dosage are for the most part, incomplete. As the psychophysiological responses and adaptations are based on the internal load, we suggest that dose is the link between the external load, internal load, and the training variables (e.g., frequency as the number of training sessions per week, density as the distribution of training sessions across a week with regard to recovery time between training sessions, and duration of a training program; Herold et al., 2019b). These variables interact with the considered training principles (e.g., overload, progression, variation, specificity, and continuity), and can be operationalized and monitored using a specific indicator (or set of specific indicators) of internal load as proxy (e.g., heart rate, blood lactate concentration, and perceived exertion; Gronwald et al., 2019; Herold et al., 2019a). According to the definitions provided, internal load as proxy of the dose could be influenced by modifying the external load by considering influencing factors (e.g., environment) and the actual state of the psychophysiological capacity level (including level of performance). In this regard, many practitioners and scientists continue to favor exercise load prescription for cardiovascular exercises and training based on percentages of maximum internal load values (percentage of maximum heart rate—%HR_MAX_ or oxygen uptake—%VO_2MAX_, heart rate reserve—%HRR). This preference is likely owing to the consistent challenges and pitfalls of individual threshold-based concepts (blood lactate thresholds—LT1, LT2 or ventilatory thresholds—VT1, VT2; Mann et al., 2013). The optimal and valid indicator(s) which are, with regard to the context and specific responses or adaptations (e.g., neuroplasticity; Budde et al., 2016b), the most suitable proxy of dosage for physical interventions is highly specific and yet-to-be-discovered. However, there is a good rationale in support of the individualization of exercise prescription by providing a distinct (comparable) dose across individuals to elicit the desired psychophysiological responses, which would in turn allow for a comparison across different individuals (Herold et al., 2019a). Finally, it is not only “genetics” but also the full range of the individual's characteristics that should be considered in an individualized exercise prescription to achieve the greatest benefit for an individual (Horwitz et al., 2013).

(iii) Thirdly, another point that warrants clarification is the relatively random recommendation to perform high intensity interval training (HIIT) to abolish non-responsiveness. The authors' recommendation for such a specific physical training regime (i.e., HIIT) is closely related to a “one-size-fits-all” approach rather than the encouragement of the individualization of physical interventions in regard of the type of physical exercise or the exercise and training variables. Similarly, interval training with moderate intensities (extensive interval training) with the aim to enhance the acute ability to respond to changes of external load could also be suitable and effective due to the factor of intermittency (Jiménez-Pavón et al., 2019). Furthermore, we do not completely agree with the provided rationale for HIIT that aims to convince the reader by claiming that a high exercise intensity [not below the second ventilatory threshold (VT2)] is necessary to increase the level of peripheral blood lactate and, in turn, the levels of the brain-derived neurotrophic factor (BDNF) because the HIIT or intensities above the VT2 are not urgently required to increase peripheral blood lactate concentration substantially (Binder et al., 2008). In this context, we must distinguish between acute responses of physical exercise (internal load) and adaptations in response to physical training. However, while we do not doubt that HIIT could be a potential strategy to increase the number of responders, this can also be true for any other physical intervention with various stimuli (continuous and intermittent regimes with different intensities) and different physical exercise modes with different motor demands (Wegner et al., 2019) (e.g., running, cycling, dancing) that follow an appropriately individualized exercise prescription. In this context, an individualized exercise prescription should, in addition to physiological factors, also address the age and personal circumstances (e.g., feasibility and concrete possibilities to be physically active, emotional and motivational situation to be physically active, type of potential activities, and possibilities to adapt and control dosage), which are bound to change over time.

In summary, to overcome the “one-size-fits-all” approach, future investigations are encouraged to pay careful attention to an appropriate use of terminology (Budde et al., 2016a), an accurate dose-response relationship (Budde et al., 2018; Gronwald et al., 2018), and a comparable dosage of physical interventions that can be induced by an individual tailored exercise prescription using internal load as proxy (Gronwald et al., 2019; Herold et al., 2019a). Speculatively, such an approach holds a greater potential to lower interindividual heterogeneity in distinct outcomes (e.g., neurocognition) which, in turn, can foster beneficial effects of physical interventions in the prevention of dementia and other neurological diseases. Additionally, we wish to stress that it is not necessary to debate whether genetic analysis contributes to the individualization of physical interventions, but rather how this information could assist the individualization of physical interventions. In this regard, when a dose-comparability across different individuals is ensured by an individualized exercise prescription, genomic analyses may provide further information on sources of interindividual heterogeneity which could, in turn, be useful to optimize subsequent individualized exercise prescriptions. Future studies in this field are required to identify the optimum trade-off between indicators of external load, training variables, and the capacity level (including level of performance) in various age groups in order to compare the effects of exercise interventions from a neurobiological perspective.

## Author Contributions

TG and HB have fully reviewed and criticized the original article, drafted the commentary, reviewed, and approved the final manuscript.

### Conflict of Interest

The authors declare that the research was conducted in the absence of any commercial or financial relationships that could be construed as a potential conflict of interest.

## References

[B1] Binder R. K. Wonisch M. Corra U. Cohen-Solal A. Vanhees L. Saner H.. (2008). Methodological approach to the first and second lactate threshold in incremental cardiopulmonary exercise testing. Eur. J. Cardiovasc. Prev. Rehabil. 15, 726–734. 10.1097/HJR.0b013e328304fed4 19050438

[B2] Budde H. Schwarz R. Velasques B. Ribeiro P. Holzweg M. Machado S.. (2016a). The need for differentiating between exercise, physical activity, and training. Autoimmun. Rev. 15, 110–111. 10.1016/j.autrev.2015.09.004 26384527

[B3] Budde H. Velasques B. Ribeiro P. Machado S. Emeljanovas A. Kamandulis S.. (2018). Does intensity or youth affect the neurobiological effect of exercise on major depressive disorder? Neurosci. Biobehav. Rev. 84, 492–494. 10.1016/j.neubiorev.2016.09.026 27693226

[B4] Budde H. Wegner M. Soya H. Voelcker-Rehage C. McMorris T. (2016b). Neuroscience of exercise: neuroplasticity and its behavioral consequences. Neural Plast. 2016:3643879. 10.1155/2016/3643879 27818802 PMC5081452

[B5] Gronwald T. de Bem Alves A. C. Murillo-Rodriguez E. Latini A. Schuetter J. Budde H. (2019). Standardization of exercise intensity and consideration of a dose-response is essential. Commentary on “Exercise-linked FNDC5/irisin rescues synaptic plasticity and memory defects in Alzheimer's models”, by Lourenco et al. published 2019 in Nature Medicine. J. Sport Health Sci. 8, 353–354. 10.1016/j.jshs.2019.03.006 31333889 PMC6620472

[B6] Gronwald T. Velasques B. Ribeiro P. Machado S. Murillo-Rodriguez E. Ludyga S.. (2018). Increasing exercise's effect on mental health: exercise intensity does matter! Proc. Natl. Acad. Sci. U.S.A. 115, E11890–E11891. 10.1073/pnas.1818161115 30568027 PMC6304946

[B7] Hawley J. A. (2008). Commentary on viewpoint: Perspective on the future use of genomics in exercise prescription. J. Appl. Physiol. 104:16253. 10.1152/japplphysiol.00047.2008 18385308

[B8] Herold F. Müller P. Gronwald T. Müller N. G. (2019a). Dose-response matters! - A perspective on the exercise prescription in exercise-cognition research. Front. Psychol. 10:2338 10.3389/fpsyg.2019.02338 PMC6839278 31736815

[B9] Herold F. Törpel A. Schega L. Müller N. G. (2019b). Functional and/or structural brain changes in response to resistance exercises and resistance training lead to cognitive improvements–a systematic review. Eur. Rev. Aging Phys. Act. 16, 10. 10.1186/s11556-019-0217-2 31333805 PMC6617693

[B10] Horwitz R. I. Cullen M. R. Abell J. Christian J. B. (2013). Medicine. (De)personalized medicine. Science 339, 1155–1156. 10.1126/science.1234106 23471391

[B11] Impellizzeri F. M. Marcora S. M. Coutts A. J. (2019). Internal and External Training Load: 15 Years On. Int. J. Sports Physiol. Perform. 14, 270–273. 10.1123/ijspp.2018-0935 30614348

[B12] Jiménez-Pavón D. Carbonell-Baeza A. Lavie C. J. (2019). Are changes in telomerase activity and telomere length due to different exercise modalities, intensity, or methods: intermittency? Eur. Heart J. 40, 3198–3199. 10.1093/eurheartj/ehz323 31132084

[B13] Kaliman P. Párrizas M. Lalanza J. F. Camins A. Escorihuela R. M. andPallàs M. (2011). Neurophysiological and epigenetic effects of physical exercise on the aging process. Ageing Res. Rev. 10, 475–486. 10.1016/j.arr.2011.05.002 21624506

[B14] Kivipelto M. Mangialasche F. Ngandu T. (2018). Lifestyle interventions to prevent cognitive impairment, dementia and Alzheimer disease. Nat. Rev. Neurol. 14, 653–666. 10.1038/s41582-018-0070-3 30291317

[B15] Lightfoot J. T. (2008). Commentary on viewpoint: perspective on the future use of genomics in exercise prescription. J. Appl. Physiol. 104, 1249. 10.1152/japplphysiol.00014.2008 18385304

[B16] Mann T. Lamberts R. P. Lambert M. I. (2013). Methods of prescribing relative exercise intensity. Physiological and practical considerations. Sports Med. 43, 613–625. 10.1007/s40279-013-0045-x 23620244

[B17] Müller P. Schmicker M. Müller N. G. (2017). Präventionsstrategien gegen Demenz. Z. Gerontol. Geriatr. 50, 89–95. 10.1007/s00391-017-1202-x 28243736

[B18] Müller P. Taubert M. Müller N. (2019). Physical exercise as personalized medicine for dementia prevention? Front. Physiol. 10:672. 10.3389/fphys.2019.00672 31244669 PMC6563896

[B19] Peck R. W. (2018). Precision medicine is not just genomics: the right dose for every patient. Ann. Rev. Pharmacol. Toxicol. 58, 105–122. 10.1146/annurev-pharmtox-010617-052446 28961067

[B20] Pescatello L. S. (2008). Commentary on viewpoint: perspective on the future use of genomics in exercise prescription. J. Appl. Physiol. 104, 1247. 10.1152/japplphysiol.01312.2007 18385302

[B21] Pickering C. Kiely J. (2019). Do non-responders to exercise exist - and if so, what should we do about them? Sports Med. 49, 1–7. 10.1007/s40279-018-01041-1 30560423 PMC6349783

[B22] Sparks L. M. (2017). *Exercise training response* heterogeneity: physiological and molecular insights. Diabetologia 60, 2329–2336. 10.1007/s00125-017-4461-6 29032385

[B23] Wegner M. Koutsandréou F. Lauterbach F. Müller-Alcazar A. Budde H. (2019). Effects of different types of exercise training on the cortisol awakening response in children. Front. Endocrinol. 10:463. 10.3389/fendo.2019.00463 31428044 PMC6689951

